# ISRIA statement: ten-point guidelines for an effective process of research impact assessment

**DOI:** 10.1186/s12961-018-0281-5

**Published:** 2018-02-08

**Authors:** Paula Adam, Pavel V. Ovseiko, Jonathan Grant, Kathryn E. A. Graham, Omar F. Boukhris, Anne-Maree Dowd, Gert V. Balling, Rikke N. Christensen, Alexandra Pollitt, Mark Taylor, Omar Sued, Saba Hinrichs-Krapels, Maite Solans‐Domènech, Heidi Chorzempa, for the International School on Research Impact Assessment (ISRIA)

**Affiliations:** 1 Agency for Health Quality and Assessment of Catalonia (AQuAS), Carrer de Roc Boronat, 81, ES-08005 Barcelona, Spain; 2 Radcliffe Department of Medicine, University of Oxford, John Radcliffe Hospital, Oxford, OX3 9DU United Kingdom; 3 The Policy Institute, King’s College London, Strand Campus, London, WC2R 2LS United Kingdom; 4 Alberta Innovates, 10104-103 Avenue NW, Edmonton, AB T5J 4A7 Canada; 5 Qatar National Research Fund, PO Box 5825, Doha, Qatar; 6 Commonwealth Scientific and Industrial Research Organisation, PO Box 883, Kenmore, Brisbane, 4069 Australia; 7 Novo Nordisk Foundation, Tuborg Havnevej 19, DK-2900 Hellerup, Denmark; 8 National Institute for Health Research, Central Commissioning Facility, Grange House 15, Church Street, Twickenham, TW1 3NL United Kingdom; 9 Fundación Huésped, Pasaje A. Peluffo 3932, Buenos Aires, C1202ABB Argentina

**Keywords:** Research impact assessment, Evaluation, Science policy, Science of science, Responsible research and innovation, Guidelines, International School on Research Impact Assessment (ISRIA)

## Abstract

As governments, funding agencies and research organisations worldwide seek to maximise both the financial and non-financial returns on investment in research, the way the research process is organised and funded is becoming increasingly under scrutiny. There are growing demands and aspirations to measure research impact (beyond academic publications), to understand how science works, and to optimise its societal and economic impact. In response, a multidisciplinary practice called research impact assessment is rapidly developing. Given that the practice is still in its formative stage, systematised recommendations or accepted standards for practitioners (such as funders and those responsible for managing research projects) across countries or disciplines to guide research impact assessment are not yet available.

In this statement, we propose initial guidelines for a rigorous and effective process of research impact assessment applicable to all research disciplines and oriented towards practice. This statement systematises expert knowledge and practitioner experience from designing and delivering the International School on Research Impact Assessment (ISRIA). It brings together insights from over 450 experts and practitioners from 34 countries, who participated in the school during its 5-year run (from 2013 to 2017) and shares a set of core values from the school’s learning programme. These insights are distilled into ten-point guidelines, which relate to (1) context, (2) purpose, (3) stakeholders’ needs, (4) stakeholder engagement, (5) conceptual frameworks, (6) methods and data sources, (7) indicators and metrics, (8) ethics and conflicts of interest, (9) communication, and (10) community of practice.

The guidelines can help practitioners improve and standardise the process of research impact assessment, but they are by no means exhaustive and require evaluation and continuous improvement. The prima facie effectiveness of the guidelines is based on the systematised expert and practitioner knowledge of the school’s faculty and participants derived from their practical experience and research evidence. The current knowledge base has gaps in terms of the geographical and scientific discipline as well as stakeholder coverage and representation. The guidelines can be further strengthened through evaluation and continuous improvement by the global research impact assessment community.

## Background

Governments, funding agencies and research organisations all over the globe increasingly seek to maximise societal and economic returns on investment in research by shaping research policy and practice. For example, in the European Union’s Horizon 2020 research and innovation programme, excellent science, industrial leadership and societal challenges are three mutually reinforcing priorities [1], and the Responsible Research and Innovation approach [2] within the ‘Science with and for Society’ programme “*aims to better align both the process and outcomes of R&I* [research and innovation]*, with the values, needs and expectations of European society*” [3]. In Canada, the Prime Minister’s mandate letter to the Minister of Innovation, Science and Economic Development stresses the importance of focusing on results that benefit Canadians [4], and the Policy on Results for all federal governmental departments sets out accountability for performance information and evaluation [5]. In Australia, the National Innovation and Science Agenda makes a commitment to “*introduce, for the first time, clear and transparent measures of non-academic impact and industry engagement when assessing university research performance*” [6]. Inevitably, the way the research process in all scientific domains is organised and funded is becoming increasingly under scrutiny. There are growing aspirations for science policy to be formulated on the basis of a scientific understanding of how science works and how to optimise its impact [7–9]. There are also important initiatives to scientifically measure and study science [10–12] as well as critical views on how research is shaped and performed [13–18].

In response to such growing demands and aspirations, the practice of research impact assessment (RIA) has been rapidly developing. Whereas interest in assessing research impact and developing evidence-based science policy is not new [19–25], early analyses mainly examined innovation processes and research outputs, such as publications, citations, and grants, using bibliometric and econometric techniques. More recently, research funders have developed interest in measuring research impact beyond academia. For example, in the case of the 2014 Research Excellence Framework (REF) of the Higher Education Funding Council for England, impact was defined as “*any effect on, change or benefit to the economy, society, culture, public policy or services, health, the environment or quality of life, beyond academia*” [26]. RIA uses a multitude of methods from social science disciplines to examine the research process with a view to maximising its societal and economic impacts such as intellectual property, spin-out companies, health outcomes, public understanding and acceptance, policy-making, sustainable development, social cohesion, gender equity, cultural enrichment, and other benefits.

In Europe, North America, Australia, and other countries around the world, RIA is already being institutionalised within national research and innovation systems. Many government agencies and research organisations are starting to use RIA as a practical tool for decision-making in scientific strategy, demonstrating accountability to research funders, or even to allocate research resources. We anticipate that the use of RIA will intensify and spread to other regions and countries.

- In the European Union, evaluations serve to create a crucial evidence base for the implementation of research and innovation programmes and are legally required for all framework programmes. Past programmes have been evaluated [27], the current programmes are being monitored [28], and based on the interim evaluation of Horizon 2020 there have been developed recommendations on how to maximise impact of future research and innovation [29]. Likewise, the League of European Research Universities recommends that universities embrace the societal impact agenda and develop transparent reward systems for all kinds of impact [30].
- Among all European countries, RIA is most developed in the United Kingdom, with practice-defining contributions ranging from the development of conceptual tools such as the Payback Framework [31–34] to the introduction of non-academic impact assessment on the national scale as in the case of the 2014 REF [26, 35, 36]. A wealth of new resources is being put in place and made openly accessible to identify and assess the impact of research both at the level of organisations and nationally [36–44], with great potential to explore methodological challenges and novel aspects of RIA such as time lags in translation [45, 46], the gender equity pathway to maximise research impact [47–49], or the relative valuation of different kinds of research impact by the general public, specific patient groups and researchers [50].
- In Spain, RIA has been used in the context of health sciences programmes [51, 52] and networks [53], with the aim to improve and test applications of various methods and frameworks. A comprehensive health research assessment system is being institutionalised by mandate of the Catalan Strategic Plan for Health Research and Innovation (PERIS). This assessment system (named SARIS) holds upon the grounds of the global lessons learned from RIA.
- In the Netherlands, the strategy is to focus on assessing the research process as a means to facilitate impact through the so-called ‘productive interactions’, i.e. “*exchanges between researchers and stakeholders in which knowledge is produced and valued that is both scientifically robust and socially relevant*” [54, 55]. Universities, funding agencies and academic organisations have jointly developed a common assessment system, the Standard Evaluation Protocol (SAP) [56], which includes relevance to society as one of the three main assessment criteria.
- In the United States, where innovation studies, research evaluation and the science of science first emerged [19], the National Science Foundation makes funding decisions on the basis of two major criteria – ‘intellectual merit’ and ‘broader impacts’ [57]. The National Institutes of Health and the National Science Foundation are leading efforts to create a repository of data and tools to assess the impact of federal investments in research called STAR METRICS® [58]. There are also many other federal and institutional efforts, such as the Evaluation of Large Initiatives project [59] and the Becker Medical Library Model for Assessment of Research [60].
- In Canada, the Canadian Academy of Health Sciences (CAHS) has adapted the Payback Framework to measure returns on investment in health research nationwide, and this has been subsequently adapted to the provincial context [61–66]. A number of national and provincial research funders have introduced assessment of ‘relevance’ [67]. In doing so, relevance is considered not only as a necessary condition for impact, but also as a value in itself [67].
- In Australia, the application of impact assessment has mainly been focused in the health domain or in sustainable development research in agriculture [68–76]. The Commonwealth Scientific and Industrial Research Organisation has developed an impact model and a case study approach spanning agriculture and fisheries, health, industry and defence, and the natural environment [77]. In line with the National Innovation and Science Agenda, a pilot was conducted in 2017 and preparations are currently underway to introduce a national engagement and impact assessment spanning all research fields in 2018. The national assessment will “*examine how universities are translating their research into economic, social and other benefits and encourage greater collaboration between universities, industries and other end-users of research*” [78].
- There is also a growing number of examples of RIA spreading to countries such as Argentina [79], Brazil [80], Guatemala [81], Hong Kong [82], Indonesia [83], Iran [84], and Qatar [85].

In designing and implementing RIA, researchers and practitioners worldwide face many challenges. Thus, developing standards and recommendations based on the systematised expert knowledge and making them openly accessible can be a compelling way to guide researchers and practitioners on how to effectively design and implement RIA. Many methodological challenges in RIA are well known to experts and have already been discussed in technical reports and policy papers [86, 87] (Box 1). Moreover, recently, there have been a number of important recommendations in peer-reviewed journals regarding different aspects of the research process. The *Lancet* series on ‘Research: Reducing Waste and Increasing Value’ [13] provides recommendations regarding the research process, including research priority setting [88]; design, conduct and analysis [89]; regulation and management [90]; inaccessible research [91]; and incomplete or unusable research [92]. The Leiden Manifesto for Research Metrics elaborates principles for metrics-based evaluation of research outputs [93] and the Metric Tide elaborates on the role of metrics in research assessment and management [94]. A manifesto for reproducible science puts forward measures to optimise the scientific process with regard to methods, reporting and dissemination, reproducibility, and evaluation and incentives [15].

Box 1 Five common methodological challenges in RIA [86]

**Table:** 

**•** **Time lags:** how do we assess the impact of research if it usually takes a long time for impact to occur? When is the right timing?
**•** **Attribution and contribution:** how do we attribute particular impacts to particular research projects and researchers (and vice-versa) if research is often incremental and collaborative?
**•** **Marginal differences:** how do we distinguish between high and low impact if there is no shared understanding of impact or assessment standards yet?
**•** **Transaction costs:** how do we ensure that the benefits of RIA outweigh its costs if the assessment process can be costly and burdensome?
**•** **Unit of assessment:** how do we determine an appropriate unit of assessment if research can be multi-disciplinary and multi-impactful?

Yet, the challenges faced during the design and implementation of RIA by practitioners based within funding organisations or institutions responsible for managing a portfolio of research are not well addressed in the current literature. We believe that standards and recommendations to guide research programme managers and other practitioners on how to effectively design and conduct RIA would prove useful both for practical applications and for establishing a common language to facilitate mutual learning in the global community of practice. Here, we propose initial guidelines by systematising expert and practitioner knowledge from designing and delivering the International School on Research Impact Assessment (ISRIA) (http://theinternationalschoolonria.com).

### Development of the ISRIA statement

ISRIA is a community of experts and practitioners from different organisations and research systems. For the past five years, we have been engaged with ISRIA in designing, delivering and applying the school’s learning programme in practice. The school was founded in 2013 based on the recognition that research programme managers and other practitioners faced challenges that were unaddressed at that time and, we believe, still are. Namely, debate on RIA lacks a focus on practitioner needs; there is a perceived mutually exclusive relationship between different methods, models and approaches; there is a nascent but too diffuse community of practice; and there is a need to build international capacity, share practice and develop standards. ISRIA recognises the growing need for practical skills and aims to fulfil it. It defines RIA as “*a growing field of practice that is interested in science and innovation, research ecosystems and the effective management and administration of research funding*” [95].

**Fig. 1:**
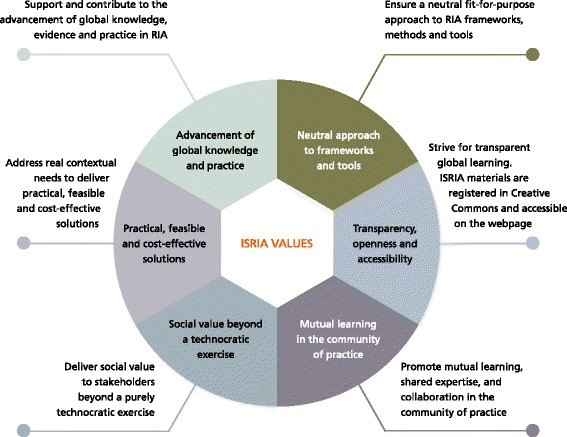
Six core values underpinning ISRIA

The school’s learning programme is underpinned by a set of six core values that guide participants to develop and implement their own RIA plan (Fig. 1). The programme stands on the recognition that, beyond technical challenges, there are also global, local, cultural and other contextual challenges. Each edition of ISRIA took place in different countries and had participants from diverse cultures and from many research disciplines. The experience and cultural competence gained through the application of the school’s learning programme in different contexts has generated a wealth of expert knowledge and practical skills that support the formulation of these guidelines.

**Fig. 2:**
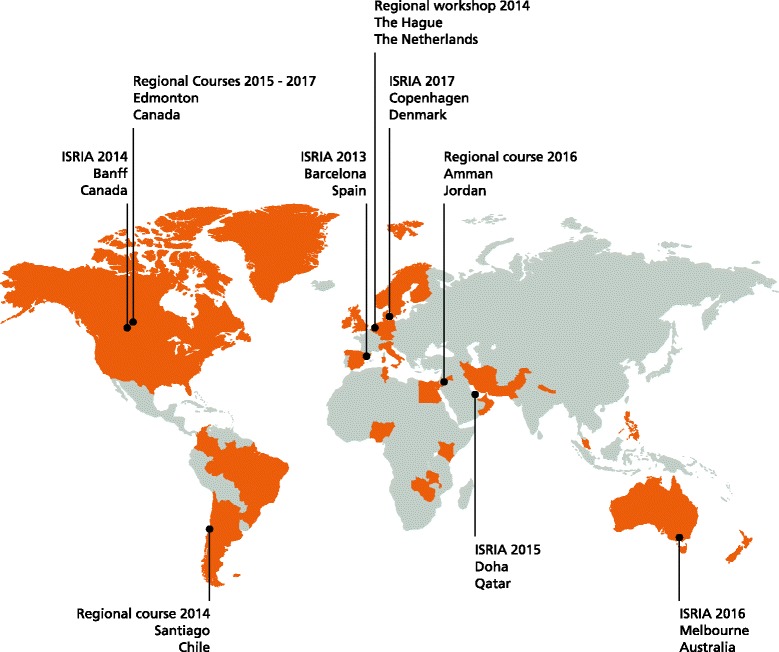
International School on Research Impact Assessment: events and participants, 2013–2017. Black dots indicate international editions, regional courses and workshops; orange areas indicate countries represented by faculty and participants

The ISRIA statement brings together insights from over 450 scholars and practitioners representing 34 countries from five continents, who participated in five international editions and seven regional ISRIA courses and workshops in 2013–2017 (Fig. 2). The largest proportion of the school’s participants come from Europe, North America, Australia, South America, and the Middle East. The best covered research area is health, followed by education, energy and environment. The highest represented stakeholders are research funding agencies, charities, government and academia.

**Fig. 3:**
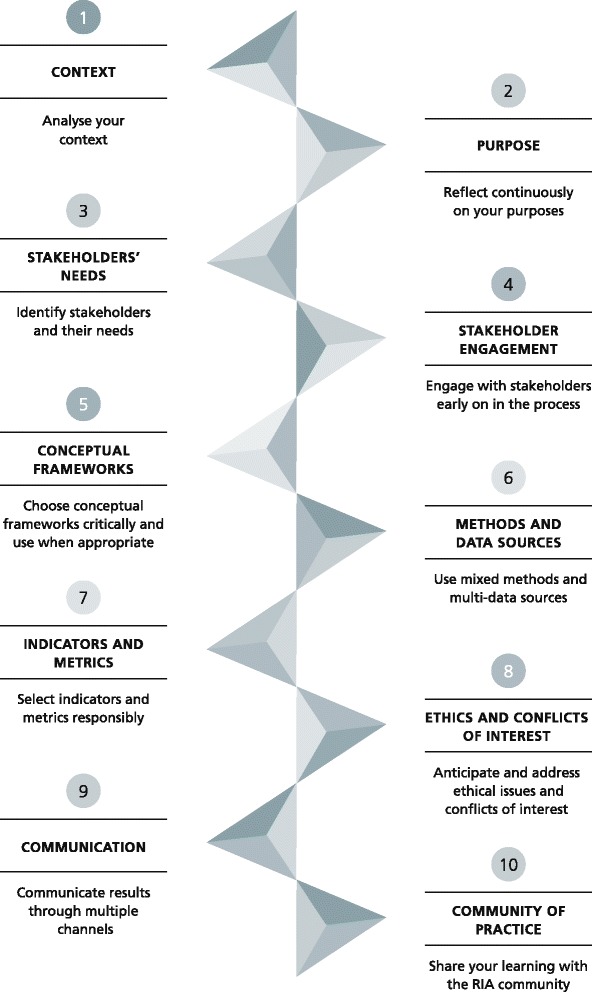
Ten-point guidelines for an effective process of research impact assessment

The authors distilled these insights into recommendations and agreed on ten-point guidelines by consensus. The ten-point guidelines relate to (1) context, (2) purpose, (3) stakeholders’ needs, (4) stakeholder engagement, (5) conceptual frameworks, (6) methods and data sources, (7) indicators and metrics, (8) ethics and conflicts of interest, (9) communication, and (10) community of practice (Fig. 3). The guidelines are oriented towards research practitioners and policy-makers in funding organisations, healthcare organisations, universities, research organisations, government agencies, industry and charities wishing to develop a process of RIA in any scientific domain and at any level of assessment.

### Ten-point guidelines for an effective process of RIA

#### 1. Analyse your context

Context analysis helps understand the internal and external environment in which research takes place and is being assessed. An enhanced understanding of the research environment illuminates why particular research is conducted, to what extent it can contribute to the wider research field, how it is relevant to the needs of potential research users, and which RIA methods and indicators to employ.

The importance of the internal research environment is such that, in the United Kingdom’s REF, it forms a major element of the overall quality profile awarded to each submission. The research environment is assessed in terms of its ‘vitality and sustainability’ using the following data and information: research strategy; staff and students; equality and diversity; research income, infrastructure and facilities; and collaboration and contribution to the discipline [26]. Analysis of the research environment can help benchmark and assess the strengths and weaknesses of the given research environment. Many countries provide detailed national data on higher education and research organisations and there are also international comparisons and rankings [96–98] that can be useful (although in interpreting these rankings it is important to understand how they have been developed and the strengths and weaknesses of the approach used).

Analysis of the external research environment can also help identify relevant macro-environmental factors and trends that may affect, or be affected by, the research undertaken in a particular country or context. These are often conceptualised as PESTLE (political, economic, social, technological, legal and environmental), STEEPLED (social, technological, economic, environmental, political, legal, ethical and demographic), or SPELIT (social, political, economic, legal, intercultural and technical) [99]. For example, while developing its own home-grown scientific and research management talent pool, Qatar’s research and development enterprise draws heavily on the international workforce and expertise. Hence, the macro-environmental factors influencing recruitment and international collaboration are particularly important for Qatar’s research and development enterprise.

#### 2. Reflect continuously on your purposes

**Fig. 4:**
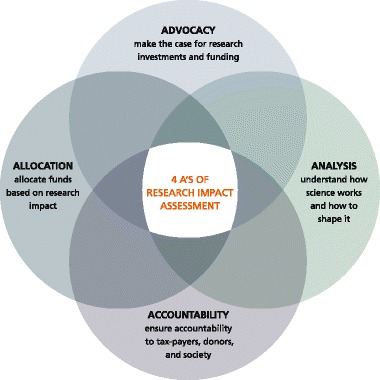
The ‘Four As’ of research impact assessment: advocacy, analysis, accountability and allocation. Adapted from [86]

Continuous reflection on the purposes of RIA and one’s relationship to the research being assessed helps refine the assessment questions and methodology. The purposes of RIA include advocacy, accountability, analysis and allocation (Fig. 4) [86]*.*

##### Advocacy

An advocacy approach to RIA is used when there is a need to ‘make the case’ for research, e.g. to demonstrate the returns of science, alleviate concerns about its value, or raise awareness and obtain more support. An advocacy approach is particularly relevant to addressing policy decision-makers when funding cycles are changing and more research investments are needed, in the context of austerity when research funding needs to be protected, or when there is a need to inform public opinion. When RIA is undertaken to demonstrate the value of research to society and how science can help grow the economy, economic return on investment approaches are usually employed to estimate impact on GDP, tax revenues, net value added, jobs created and other returns. For example, a number of United Kingdom studies focus on the internal rate of return, including spill-overs, time-lag, percentage of attribution and health gain (monetarised measures of quality of life gained), net savings for the health system and net health gains [37–40].

##### Analysis

A robust analytical approach should ideally underpin all other ‘A’s’ , particularly when an understanding of how science works is required in order to optimise its returns. This often involves understanding the barriers to and facilitators of impact, identifying dysfunctions within research programmes, as well as highlighting opportunities to add more value to research during its planning and execution. For example, a series of ‘Retrosight’ studies in different fields of health research has used detailed case studies to examine how individual pieces of research generate different kinds of impact over a 10–20 year timeframe, as well as characteristics of projects, teams and institutions that are associated with that impact [100–102]. Key lessons that emerged from these studies included the importance of engaging with non-academic stakeholders during the research, and the value of particular skills in a research team such as working across boundaries and being able to think strategically about pathways to impact [103].

##### Accountability

An accountability approach to RIA is used to ensure accountability to tax-payers, donors and society for research funding. With the increasing pressure to reduce public spending, there is a greater emphasis on transparency, efficiency, value to the public and a return for the investment made by the public, private and charitable sectors in research. For example, Australia’s national research evaluation framework, Excellence in Research for Australia (ERA) [104], is considered to be “*one of the primary mechanisms that Government, public and private sectors have to account for their expenditure on higher education research sector*”. An independent review of the benefits of ERA found that, while improving accountability, transparency and policy-making, ERA helps to increase the social rate of return from research, generate cost savings, increase university revenue and enhance economic activity [105].

##### Allocation

An allocation approach to RIA is used to incentivise research excellence by providing economic rewards through the allocation of resources. Allocation of resources is generally based on the assessment of several dimensions of research quality, impact and environment using explicit criteria. In the United Kingdom, non-academic impact was included in the national REF process for the first time in 2014, with the weighting of 20% for non-academic impact, meaning that 20% of approximately £1.6 billion of quality-related research funding allocated to higher education institutions annually is allocated on the basis of non-academic impact.

#### 3. Identify stakeholders and their needs

Attention to stakeholders and their needs is important for the success of any RIA. Identifying and analysing stakeholders and their needs helps prioritise stakeholder interests, develop engagement strategies and determine RIA requirements.

Stakeholders are the people and organisations with an interest in the outcome of a given RIA. For RIA to influence practice, it needs to address stakeholders’ interests, beliefs and behaviour. This is particularly true in the public sector, where “*‘success’ for public organisations – and certainly survival – depends on satisfying key stakeholders according to their definition of what is valuable*” [106]. Stakeholders’ needs are further influenced by their country’s or community’s cultural values, associated with the geographical location, traditions, language and religion, by political and organisational rules of behaviour, and by personal socio-demographic characteristics.

Different stakeholders play different roles in the research process, operate in different contexts, possess different types of information, and therefore value different aspects of RIA. For example, according to their role in the research process, stakeholders can be classified into research funders, research participants, researchers, research users and research beneficiaries. Whereas research funders are usually concerned about demonstrating an effective use of resources, improving resource allocation and formulating evidence-based science policy, researchers are usually interested in demonstrating research outputs and impacts over time, promoting personal or institutional research agendas, and making the case for new resources.

**Fig. 5:**
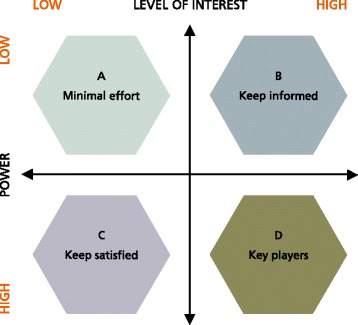
Power versus interest grid – the Mendelow matrix. Adapted from [110]

Various theoretical approaches can be used to identify stakeholders, determine their salience, and prioritise the levels of attention that they require in the RIA process. For example, stakeholders’ salience can be determined on the basis of their power, the legitimacy of their relationship and the urgency of their claim [107]. Stakeholder analysis and prioritisation can be further assisted by the use of power versus interest grids, stakeholder influence diagrams, problem-frame stakeholder maps, and the participation planning matrix [106, 108, 109]. A power versus interest grid, also known as the Mendelow matrix [110], is one of the most frequently used methods of stakeholder analysis (Fig. 5). The information required for stakeholder analysis can be gathered from organisation- and programme-level strategic plans, annual reports, governance and management board papers and minutes, websites, surveys, interviews, previous evaluations and other documents.

#### 4. Engage with key stakeholders early on

Engaging with stakeholders early and throughout the process of RIA can help ensure the social robustness of RIA and make real advances in how science is shaped. Developing interpersonal engagement skills and cultural competence can further facilitate an effective translation of RIA into practice.

It is argued that, in recent decades, a social contract between science and society has been redrawn to include not only production of scientifically reliable knowledge by scientists (Mode 1), but also a transparent and participatory process of knowledge production characterised by researchers’ engagement with research users and other stakeholders during research design and implementation (Mode 2) [111–113]. Such Mode 2 knowledge production is likely to result in ‘socially robust’ knowledge and therefore more effective translation of knowledge into practice [112]. Mode 2 knowledge production is also more efficient because it presupposes direct adoption of research findings and innovations and requires less dissemination and knowledge mobilisation.

Many research funders promote engagement with stakeholders as a means of co-creating and enhancing future research impact at different stages of the research process, including design, implementation and evaluation. For example, the European Union’s Responsible Research and Innovation approach aims to engage all societal actors in the research and innovation process [3]. The National Institute for Health Research (NIHR) in England promotes involving patients and the public in health research, not only as research participants, but also as research users “*advising* [NIHR] *about what research should be funded and helping to design research studies*” [114]. The assessment system SARIS in Catalonia includes, hand-in-hand with the evaluation, engagement with stakeholders as a means of enhancing research impact [115].

Effective translation of RIA into practice can be further facilitated by developing interpersonal engagement skills and cultural competence. Knowing one’s and one’s teams’ preferences and biases well is as important as knowing how to engage with stakeholders from diverse cultures and backgrounds who may have a different set of values, preferences and biases. Interpersonal skills and cultural competencies are required to engage with such stakeholders without compromising the robustness and rigour of RIA. According to the American Evaluation Association, “*cultural competence requires awareness of self, reflection on one’s own cultural position, awareness of others’ positions, and the ability to interact genuinely and respectfully with others*” [116].

#### 5. Choose conceptual frameworks critically

Conceptual frameworks can support RIA by reducing the complexity of the phenomenon under investigation for the purposes of data collection, organisation and analysis. Frameworks can also help address major methodological challenges and comparisons of research impact across different disciplines, institutions and countries.

Research impact is a complex and often unpredictable phenomenon, which makes the task of assessing it difficult [117]. Conceptual frameworks can help make this task easier in a number of ways. First, RIA practitioners can use readily available conceptual frameworks to reduce the complexity of the phenomenon under investigation for the purposes of data collection, organisation and analysis*.* Second, frameworks can help address major methodological challenges of RIA, such as attribution (assigning the right impacts to a specific piece of research or vice versa), time-lag (determining the time for impact and the right timing to engage in a RIA) and the counterfactual (examining what would have happened if the given piece of research did not occur). Third, frameworks can allow transparent, longitudinal and quantifiable comparisons of research impact across different disciplines, institutions and countries. Finally, frameworks can facilitate communication of the results of RIA to stakeholders and the public in a clear and accessible manner. Yet, because frameworks deliberately reduce the complexity of the phenomenon under investigation, they need to be selected critically and transparently.

As stated in the founding values (Fig. 1), ISRIA does not advocate for any specific framework, but recommends to critically choose frameworks in a way that fits the context and purpose of a given RIA exercise and to explicitly state the limitations of the chosen framework. There are a number of literature reviews to help practitioners understand the advantages and limitations of different conceptual frameworks and approaches [87, 117–121]. For example, the Payback Framework has been widely used for an understanding of the research process and pathways to impact in the United Kingdom and many other countries. The CAHS model [62], an adaptation of the Payback Framework, has been widely used in Canada because it aims to provide consistency and comparability while remaining flexible for interpretation at different levels tailored to the Canadian context. The CAHS model has also been used in Spain for communication, advocacy and formative purposes [51, 52].

#### 6. Use mixed methods and multi-data sources

RIA is best approached using a combination of mixed methods and a variety of data sources. Triangulating methods and data sources can enhance the robustness and trustworthiness of the assessment.

Unlike basic science, which strives to conduct valid and reliable research with generalisable findings, RIA strives to understand research impact from the perspectives of certain stakeholders. Given the applied nature of RIA, the value of RIA can be increased by enhancing the robustness of methods and data as well as ensuring the trustworthiness of findings and recommendations. An effective way to do so is to triangulate different methods and data, i.e. to use more than one method and data source to develop rich accounts of research impact. If these accounts point to the same result, then it is deemed to be trustworthy. RIA practitioners are not expected to be experts in all methods, but need to understand the advantages and disadvantages, scope and limitations of different methods in order to gather data and choose methods that address the stakeholder assessment questions in the most effective and efficient way.

Different design approaches have different strengths and weaknesses, and the selection of methods imply trade-offs between structured and purposive designs, stratified and random experiments [122]. Case studies provide powerful narratives that can be easily understood by stakeholders and the general public, but on the other hand they are costly, time consuming, burdensome and might be perceived as subjective. Surveys allow the collection of a large amount of data from a wide range of stakeholders with a relatively low burden on key informants, but potential limitations include sampling errors, low response rates and inadequate questionnaire validation in different contexts. Bibliometric approaches are well established, have a broad range of data sources available and are capable of providing robust quantitative analysis, but caution must be exercised in using non-normalised indicators, especially outside natural and health sciences. Further, they also tell us little about impact beyond academia.

In choosing the appropriate mix of methods, practitioners face the crucial decision of what methods to mix and how many of them. The number of methods is usually determined by the number of questions requiring different data and by the saturation point when adding more methods does not improve triangulation results. Before collecting data ex novo, it is important to map available internal data and external sources. Conducting complex analyses in-house might not be cost effective as certain types of analysis, particularly those requiring specialist technical expertise (e.g. bibliometrics or economic returns), can be effectively contracted out. It is also important to consider cost implications and practicality issues to ensure RIA is affordable, cost-effective and efficient. Finally, it might be relevant to analyse whether the potential results can address the type of messages expected or needed by stakeholders and end-users of the research.

#### 7. Select indicators and metrics responsibly

The misuse of quantitative indicators and metrics can lead to gaming and unintended negative results. Any quantitative indicators and metrics need to be used responsibly relative to the context and in support of other types of evidence. While using specialised methodologies such as bibliometrics or econometrics, it is recommended to use critical recommendations from experts.

A key concern for measuring impact is reflected in a statement attributed to Albert Einstein, “*not everything that counts, can be counted*”, in other words, focusing on what is measurable rather than what is important; this is particularly relevant when taking account of the context of each given impact assessment. On the other hand, indicators and metrics can be used to think through what counts as evidence, demonstrating whether impact occurred or not. Indicators provide signals of impact, but do not provide comprehensive assessment of the full range or the many factors that contributed to those impacts. The desire is to use indicators and metrics as one line of evidence to make better decisions. Quantitative measurement misuse can lead to unintended negative results such as the pressure to ‘publish or perish’ at all costs or excessive self-citation in research. To avoid such unintended behaviours, indicators and metrics need to be selected responsibly. Namely, it is recommended that a balanced set (menu) of indicators and metrics are used to answer the stakeholder assessment questions that focus on their impacts of interest.

Measuring impact is a practice that requires measurement expertise and a transparent participatory process to ensure that recommended indicators are valid, reliable and socially robust. Using a mix of quantitative and qualitative measures can help understand the ‘what’ but also the ‘how’ and ‘why’ impacts occurred. As was noted in the recent Metric Tide report on the role of metrics in research assessment, “*carefully selected indicators can complement decision-making, but a ‘variable geometry’ of expert judgement, quantitative indicators and qualitative measures that respect research diversity will be required*” [94]. Indicator expert panels and Delphi surveys [123] can be used to take into account the opinions of a diverse sample of experts in the selection of the best impact indicators and metrics. Involving lay members of the public, stakeholders and research end-users in the development and selection of indicators can increase the social robustness of selecting indicators as well as provide a balanced set of perspectives. Selecting sets of indicators and metrics that conform to best practice criteria, such as Focused, Appropriate, Balanced, Robust, Integrated, Cost Effective (FABRIC), will also help ensure proper use and quality [124]. Finally, the many cautions for measuring impact can be addressed by establishing mitigating strategies prior to implementation (Table 1).

**Table 1:** Measurement cautions and mitigating strategies

Cautions	Mitigating strategies
• Only selecting available indicators	• Identify a menu of aspirational indicators and data sources
• Measuring too many things	• Select a key set of indicators
• Using only lagging indicators	• Balance with leading indicators
• Double counting	• Look at contributions from different stakeholders
• Focusing on the indicator	• Focus on the programme change

Alberta Innovates provides a case example of integrating measurement into its impact assessments. It is a Canadian-based, publicly funded provincial research and innovation funding organisation mandated to improve the social and economic well-being of Albertans. It uses a standardised Research to Impact Framework for health sciences that guides the selection of indicators and use of mixed methods in impact assessment [66]. Assessments are conducted at the programme, portfolio, organisational and system levels. The framework was designed to answer stakeholder questions and identify their impacts using the five CAHS impact categories. A mixed methods and multi-data source approach is used to assess the impacts of its investments across the funding cycle. Impact measures are collected annually, additional indicators and measures are collected through annual scheduled evaluations, and impact case studies are conducted retrospectively in assessing and communicating impact. Table 2 provides a sample of impact indicators.

**Table 2:** Sample of impact indicators in health research

Impacts	Indicators
Capacity-building	Leveraged funding, research tools and methods, use of facilities and resources, career trajectory of researchers
Advancing knowledge	Bibliometrics, engagements, esteem measures, collaborations and partnerships
Informing decision-making	Influence on policies, practices, products, processes and behaviours (both in health and the determinants of health)
Health	Medical and health interventions, health quality indicators, health status
Economic and social benefits	Intellectual property and licensing, spin outs, economic returns, jobs, economic diversity and productivity
Social engagement	Public involvement, dissemination, engagement with relevant patient or commissioning groups, culture and creativity

Recently, there has been an explosion of commercially available tools and platforms for reporting impact metrics in a standardised manner, with many research funders developing their own. In choosing between readily available reporting tools and developing new ones, the following criteria can be used to evaluate their effectiveness against the ideal system proposed by Wooding et al. [125]:

- Capturing the full range of impact and benefits;
- Allowing aggregation of impacts as well as their disaggregated reporting;
- Valuating different types of impacts in a common currency;
- Ensuring a low burden on researchers and having low administration costs;
- Capturing and comparing information fairly across different grants or types of research.
- Providing timely information while allowing time for impact to occur.

#### 8. Anticipate and address ethical issues and conflicts of interest

Undertaking RIA and implementing its recommendations may raise ethical issues and create conflicts of interest at both personal and organisational levels. Anticipating and addressing such ethical issues and conflicts of interests can help maximise the social value of RIA.

When contacting researchers and organisations for information, it is important to state the purpose of RIA and to consider how they may perceive the RIA practitioner’s aims and intentions. Researchers may often be asked to report back to funders on the results of their work; if their information is likely to affect future funding, they should be made aware of this. Nevertheless, even if RIA is carried out independently with no implications for further funding, this reassurance should also be made explicit. Moreover, RIA practitioners should be mindful of the burden they may place on researchers and organisations while emphasising the importance and need for them to report accurately and comprehensively for good quality RIA studies to take place. A significant commitment of time and effort to provide the required information or to participate in RIA studies may also create conflict of commitment.

Academic or professional recognition, funding and other direct, indirect, actual or potential personal and organisational benefits associated with certain RIA projects and commissions may raise further ethical issues and create perverse incentives for biased assessment in favour of RIA commissioners or some other stakeholders that run counter to the public good. Therefore, undertaking RIA purely as a technocratic exercise may lead to conflicts of interests between individuals and organisations undertaking it on the one hand, and the wider society on the other. In line with the values of ISRIA, such conflicts of interest should be anticipated and addressed to maximise the social value of RIA. Given that RIA is undertaken by individuals from different organisations and professions, in anticipating and addressing ethical issues and conflicts of interest, RIA practitioners should follow their organisational and professional ethical regulations and codes of practice, exercise their personal judgement and be aware of their own personal cognitive biases.

Different organisations and professions have different ethical regulations and codes of practice for disclosing and avoiding conflicts of interest with regard to employment, funding, remuneration, hospitality, consultancy, intellectual property, paid governance and advisory roles, paid membership of speakers panels, and other benefits that may impair the objectivity and impartiality of RIA as well as create conflicts of commitment. For example, the American Evaluation Association considers honesty/integrity and responsibilities for general and public welfare as some of the most important guiding principles for the profession of evaluation, in particular with regards to the scope of evaluation and its results, costs, methodological limitations, changes to the project plans, objectivity, underlying interests and values, freedom of information, and maintaining a balance between client needs and other needs [126].

#### 9. Communicate results through multiple channels

A comprehensive and diversified communication strategy can facilitate effective translation of RIA results into practice. Different stakeholders can be reached most effectively using different communication channels and messages tailored according to their needs and knowledge uptake capacities.

Effective translation of RIA results into practice depends on the effective communication strategy and skills as much as anything else. Understanding how different stakeholders are best approached and tailoring messages based on RIA results according to their needs, context and preferred communication means is imperative. Whereas a detailed RIA report may be an effective communication strategy for one group of stakeholders, others would better appreciate a summary of the key messages in lay terms, an executive summary, or a peer-reviewed academic publication. For example, to support active public involvement in health research, the United Kingdom national advisory group INVOLVE recommends that all research applications and reports include a plain English summary – “*a brief summary that has been written for members of the public and an interested audience rather than specialists… clearly and simply, without jargon and with an explanation of any technical terms that have to be included*” [127]. Even within the RIA community it is worth noting that terms may be used differently from country to country or in different research disciplines, making it important to carefully define concepts and terms and avoid overly-technical language as far as possible.

Communication is also crucial for an effective engagement throughout the RIA process. Communication of your RIA plan, process and findings is likely to be strengthened by the use of visualisation tools such as infographics, diagrams, charts and other visual aids. A recent example of innovative visualisation of RIA results includes infographics, alluvial and chord diagrams, word clouds, heat maps and impact wheels, and synthesising complex data to reveal where research has had a societal impact [36]. With increasing use of the web and social media by research stakeholders, impact assessment results can be rapidly communicated through a variety of media, including research blogs, social networks and web feeds. Such communication channels are also useful tools for establishing and maintaining international networks, supporting collaboration, and building communities of practice around particular areas and approaches.

#### 10. Share your learning with the RIA community

Major scientific advances and impact on policy and practice are often achieved through the mutual learning of scholars and practitioners. For RIA to continue developing its methods and grow its evidence base, it is important for scholars and practitioners to share their learning with the RIA community of practice.

As a multidisciplinary field of practice, RIA is sustained by empirical knowledge and practical skills of the community of practice. The latter is “*a group of people who share a concern, set of problems, or a passion about a topic, and who deepen their knowledge and expertise in this area by interacting on an ongoing basis*” [128]. Sharing learning with the RIA community of practice can be done in several ways. First, publication in peer-reviewed journals allows shared learning and ensures its quality and trust-worthiness through peer-review. Open access publication has the additional benefits of increased visibility, citation, usage and attention [129]. Second, participation in conferences, workshops and other similar events allows quicker sharing of learning as well as the development of trust and collaboration between event participants. Third, the internet provides the quickest opportunity to share knowledge and data through websites and blogs as well as to establish collaboration between scholars and practitioners across many countries through social networks (https://www.linkedin.com/groups/5180935) and social media (https://twitter.com/resimpactschool). Finally, professional associations such as the American Evaluation Association (http://www.eval.org/), professional societies such as the International Society for Scientometrics and Informetrics (http://issi-society.org), and professional training and development networks such as the ISRIA (http://theinternationalschoolonria.com/) offer platforms for interaction and mutual learning through regional or international courses, conferences, workshops and thematic groups where practitioners can discuss and learn about specific topics.

## Conclusions

The guidelines can help practitioners improve the process of RIA, but they are by no means exhaustive and require evaluation and continuous improvement. The prima facie effectiveness of the guidelines is based on the systematised expert and practitioner knowledge of the school’s faculty and participants derived from their practical experience and research evidence. The current knowledge base has gaps in terms of geographical and scientific areas as well as stakeholder coverage and representation. With that in mind, we invite readers to put these guidelines into practice, develop them further, and strengthen them through evaluation and continuous improvement. We also encourage the sharing of experience and cultural competence gained through implementing these guidelines in new contexts. In doing so, we hope these guidelines facilitate the further development of a global RIA community of practice.

## Abbreviations

CAHS: Canadian Academy of Health Sciences
ERA: Excellence in Research for Australia
ISRIA: International School on Research Impact Assessment
NIHR: National Institute for Health Research
REF: Research Excellence Framework
RIA: Research Impact Assessment
SARIS: Sistema d’Avaluació de la Recerca i Innovació en Salut (System of Evaluation of Health Research).
